# The impact of clinical phenotypes of coronary artery disease on outcomes in patients with atrial fibrillation: A post‐hoc analysis of GLORIA‐AF registry

**DOI:** 10.1111/eci.14378

**Published:** 2025-01-13

**Authors:** Bi Huang, Yang Liu, Ho Man Lam, Hironori Ishiguchi, Tze‐Fan Chao, Menno V. Huisman, Gregory Y. H. Lip, the GLORIA‐AF Investigators

**Affiliations:** ^1^ Liverpool Centre for Cardiovascular Science at University of Liverpool Liverpool John Moores University, and Liverpool Heart & Chest Hospital Liverpool UK; ^2^ Department of Cardiology The First Affiliated Hospital of Chongqing Medical University Chongqing China; ^3^ The Second Affiliated Hospital of Nanchang University Nanchang China; ^4^ Division of Cardiology, Department of Medicine and Clinical Science Yamaguchi University Graduate School of Medicine Ube Japan; ^5^ Division of Cardiology, Department of Medicine Taipei Veterans General Hospital Taipei Taiwan; ^6^ Institute of Clinical Medicine and Cardiovascular Research Center National Yang Ming Chiao Tung University Taipei Taiwan; ^7^ Department of Thrombosis and Hemostasis Leiden University Medical Center Leiden The Netherlands; ^8^ Department of Clinical Medicine Aalborg University Aalborg Denmark; ^9^ Medical University of Bialystok Bialystok Poland

**Keywords:** atrial fibrillation, cardiovascular events, coronary artery disease, novel oral anticoagulant drugs

## Abstract

**Background:**

Coronary artery disease (CAD) and atrial fibrillation (AF) often coexist, but the impact of clinical phenotypes of CAD on outcomes in AF patients in the non‐vitamin K antagonist oral anticoagulant drugs (NOACs) era is less well understood.

**Methods:**

This was a post‐hoc of the GLORIA‐AF registry, a global, multicenter, prospective AF registry study. Patients were divided into three groups: prior history of myocardial infarction (MI)/unstable angina group (Group 1); stable angina group (Group 2); and a control group without stable angina or history of MI/unstable angina. The primary endpoint was the composite of all‐cause death or stroke, and the safety endpoint was major bleeding.

**Results:**

A total of 24,827 patients were included in this analysis (median age was 71 (IQR, 64–78) years; 55% male) and 5394 (21.7%) had CAD. During a follow‐up of 2 years, the incidence of the primary endpoint was 5.99 (95% CI, 5.33, 6.71) per 100 patient‐years in Group 1, 4.04 (95% CI, 3.55, 4.70) per 100 patient‐years in Group 2, and 2.79 (95% CI, 2.62, 2.96) per 100 patient‐years in the control group (*p* < .001). Compared the control group, the adjusted hazard ratio of the primary composite endpoint in Groups 1 and 2 were 1.58 (95% CI, 1.37, 1.83, *p* < .001) and 1.22 (95% CI, 1.04, 1.43, *p* = .012), respectively. Among anticoagulated patients with AF and CAD, NOACs were associated with a reduced risk of the primary composite endpoint and major bleeding, compared with vitamin K antagonists (VKA).

**Conclusions:**

CAD was prevalent in patients with AF, and clinical phenotypes of CAD influenced outcomes in patients with AF, with a history of MI/unstable angina being associated with a significantly increased risk of CV events, compared to stable angina. NOACs were superior to VKA in terms of the effectiveness and safety outcomes in patients with AF and concomitant CAD.

## Graphical abstract

Clinical phenotypes of coronary artery disease were associated with prognosis in patients with atrial fibrillation, with stable angina patients faring better than those with a history of myocardial infarction/unstable angina. NOACs outperformed VKA in terms of primary endpoint and safety endpoint in patients with atrial fibrillation and concomitant coronary artery disease.

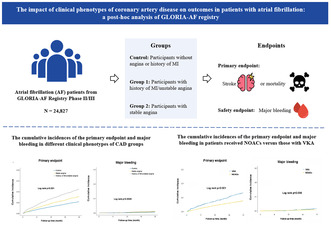

## 1 INTRODUCTION

Coronary artery disease (CAD) is the most common cardiovascular disease globally^1^ and atrial fibrillation (AF) is the most common arrhythmia in clinical practice.^2^ Both have close relationship and often coexist; the prevalence of CAD in patients with AF is reported from 17% to 59.4%.^3^, ^4^ In the setting of myocardial infarction (MI), about 10% of patients have AF and nearly 25% of patients without prior AF would develop AF at or after MI.^5^ Previous studies have shown AF increases the risk of poor prognosis in patients with CAD^6^, ^7^, ^8^; however, the impact of CAD phenotypes on outcomes in patients with AF is less understood.

Steensig et al.^4^ demonstrated that underlying coronary disease was strongly associated with elevated risk of thromboembolism beyond the components of the CHA_2_DS_2_‐VASc score, which highlighted the prognostic importance of non‐MI CAD in AF patients. Hence, angiographically significant CAD was included as a component of “V” criteria in the CHA_2_DS_2_‐VASc score for stroke risk stratification.^9^, ^10^, ^11^ Nevertheless, the clinical phenotypes of CAD vary significantly, ranging from asymptomatic to clinical angina and MI. It is currently unclear whether the cardiovascular event risks differ among patients with different clinical phenotypes of CAD combined with AF in the non‐vitamin K antagonist oral anticoagulant drugs (NOACs) era.

Accordingly, this study aims to evaluate the characteristics, treatment, and prognosis of different clinical phenotypes of CAD in patients with AF through a post‐hoc analysis of the GLORIA‐AF registry. Second, we examined the impact of sex, ethnicity and concomitant antiplatelet use on outcomes.

## 2 METHODS

### 2.1 Study design

The design of the GLORIA‐AF registry (https://clinicaltrials.gov/ct2/home, NCT01468701, NCT01671007, and NCT01937377) has been previously published.^12^ In brief, GLORIA‐AF is a prospective registry of patients with newly diagnosed AF at risk for stroke and involved five geographical regions all over the world.^12^ Patients were enrolled consecutively in a broad variety of sites in Asia, Europe, North America, Latin America, and Africa/Middle East (Africa/Middle East did not enrol patients in Phase III).

The GLORIA‐AF registry included three overlapping phases. Phase I was the period before NOACs were available; Phase II began when dabigatran was approved in each participating country (2011); Phase III began when propensity score comparisons indicated a substantial overlap in the range of the scores for those receiving dabigatran and those receiving vitamin K antagonist (VKA) to facilitate a valid assessment of safety and effectiveness (2014). The present study analysed data enrolled in Phase II and Phase III.

### 2.2 Inclusion and exclusion criteria in GLORIA‐AF

Inclusion criteria were as follows: adult patients, new‐onset (<3 months before enrollment, in Latin America <4.5 months) non‐valvular AF, and ≥1 risk factor for stroke in CHA_2_DS_2_‐VASc score. Main exclusion criteria were as follows: mechanical heart valves or valve disease requiring surgical valve replacement, history of oral anticoagulation with VKA less than 60 days for any indication, generally reversible cause of AF, an indication other than AF for VKA treatment and life expectancy less than 1 year. Ethics approval was obtained from the local institutional review board. Enrolment required informed consent and the study complies with the Declaration of Helsinki.

### 2.3 Data collection in GLORIA‐AF

An electronic data capture system was used to collect and store data. High levels of data integrity were ensured by in‐person monitoring, bimonthly calls to all sites to review data quality, entry and open queries, biweekly updates of follow‐up on data trends, regular visits by audit teams as well as quarterly medical review meetings to assess aggregate data.

### 2.4 Study endpoints

The primary endpoint was a composite of all‐cause death or stroke, and the secondary endpoint was all‐cause mortality and major adverse cardiovascular events (MACE), including cardiovascular (CV) death, stroke, and MI during the 2‐year follow‐up period. The exploratory endpoint was the individual components of MACE. The safety endpoint was major bleeding.

Based on the patients' medical history and clinical presentation at enrollment, they were divided into three groups: history of MI/unstable angina group (Group 1), stable angina group (Group 2), and control group without angina or history of MI. The outcomes among the three groups were compared, and then patients received oral anticoagulants (OAC) were further selected for analysis of the efficacy and safety of NOACs versus VKA.

### 2.5 Statistical analysis

The baseline characteristics of the patients were presented with interquartile range for continuous variables and compared by Wilcoxon signed rank test. Categorical variables were presented as number and percentage, and compared by Pearson chi‐square test.

Kaplan–Meier (K‐M) survival curves were constructed to analyse the cumulative incidence of events and were tested with log‐rank test. Multivariate Cox regression models were used to analyse the association between different clinical phenotypes of CAD and the outcomes. The variables included in the Cox regression model comprised those with statistical significance at baseline or those without statistical differences but supported by theory or literature as being related to prognosis. Additionally, variables with significant collinearity are selected based on clinical judgement to ensure the appropriate variables were included in the model. Initially, variables associated with prognosis are preliminarily screened through univariate Cox regression. Variables with *p* < .1 and those with *p* > .1 but previously reported to be related to prognosis were included in the multivariate Cox regression model. Independent factors associated with the study endpoint were then determined using the stepwise regression method. The models were corrected for age, sex, body mass index, comorbidities (hypertension, heart failure, left ventricular hypertrophy, diabetes, chronic obstructive pulmonary disease, previous transient ischemic attack [TIA]/stroke), type of AF, EHRA score, creatinine, systolic blood pressure, heart rate, CHA_2_DS_2_‐VASc score, HAS‐BLED score, and medications (aspirin, NOACs [dabigatran, rivaroxaban, apixaban, edoxaban], VKA, beta blockers, digoxin, angiotensin‐converting enzyme inhibitor, angiotensin receptor blocker, statins and diuretics).

The associations of OAC (NOACs vs. VKA) with the outcomes in patients with CAD were also analysed by multivariate Cox regression and adjusted for age, sex, body mass index, comorbidities (hypertension, heart failure, left ventricular hypertrophy, diabetes, chronic obstructive pulmonary disease, previous TIA/stroke), type of AF, EHRA score, creatinine, systolic blood pressure, heart rate, CHA_2_DS_2_‐VASc score, HAS‐BLED score and medications (aspirin, beta blockers, digoxin, angiotensin‐converting enzyme inhibitor, angiotensin receptor blocker, statins and diuretics).

Subgroup analyses were used to evaluate the effect of NOACs versus VKA on the primary endpoint across different subgroups, including sexes (male and female), ages (<75 years and ≥75 years), races (White, Asian, and others), regions (Asia, Europe, North America, Latin America and Africa/Middle East), use of aspirin or not, concomitant of heart failure or not, concomitant of hypertension or not, concomitant of diabetes or not, and concomitant of CKD or not.

In sensitivity analysis, a 1:1 propensity score matching (PSM) with logistic regression was performed to compare the impact of NOACs and VKA on outcomes. We performed a greedy nearest neighbour matching model to balance the baseline characteristics and an absolute standardized mean difference (SMD) between NOACs and VKA group lower than .1 was considered well‐matched.

The adjusted hazard ratios (HRs) with their respective 95% confidence intervals (CIs) were calculated. All statistical tests were two‐tailed, and *p* < .05 was considered to be statistically significant. All statistical analyses were carried out using RStudio (Version 1.3.1093).

## 3 RESULTS

### 3.1 Baseline characteristics

A total of 24,827 patients [median age: 71 (IQR 64–78); 55% male] with complete data in GLORIA‐AF phase II and phase III were included in this analysis. The median follow‐up time was 2 years. Among the 24,827 patients, 5394 (21.7%) had CAD, of which 2717 had a history of prior MI/unstable angina (Group 1) and 2677 had stable angina (Group 2).

Table 1 shows the baseline characteristics among the three groups of patients. Compared with patients in the control group, patients in Group 1 and Group 2 were older (median 73 years in both groups vs. 70 years in control group, *p* < .001) and more likely to be male (70% in Group 1 vs. 61% in Group 2 vs. 53% in control group, *p* < .001). Group 1 had relatively lower proportion of white patients (73%) but had relatively higher proportion of Asian patients (21%) compared with other two groups. Patients in Group 1 and Group 2 had significantly more comorbidities, including hypertension, diabetes, heart failure, hyperlipidaemia, left ventricular hypertrophy, peripheral artery disease, chronic kidney disease, chronic obstructive pulmonary disease, history of TIA/stroke and bleeding (all *p* < .001). Paroxysmal AF was more prevalent in Group 1 and Group 2 (60% in Group 1 vs. 59% in Group 2 vs. 54% in control group, *p* < .001).

**TABLE 1:** Baseline characteristics of the patients.

	Overall patient (*N* = 24,827)	Control (*N* = 19,433)	Stable angina (*N* = 2677)	History of MI/unstable angina (*N* = 2717)	*p* Value
Age (years)	71 (64, 78)	70 (63, 77)	73 (67, 79)	73 (66, 79)	<.001
Sex (male, %)	13,730 (55%)	10,217 (53%)	1621 (61%)	1892 (70%)	<.001
Race (*n*, %)					
White	18, 515 (75%)	14, 516 (75%)	2, 208 (76%)	1, 917 (73%)	.034
Asian	4, 602 (19%)	3, 564 (18%)	479 (18%)	559 (21%)	
Others	1, 710 (6.9%)	1, 353 (7.0%)	170 (6.4%)	187 (6.9%)	
Region (*n*, %)					
Asia	4, 583 (18%)	3, 452 (18%)	556 (21%)	575 (21%)	<.001
Europe	12, 353 (50%)	10, 003 (51%)	1, 091 (41%)	1, 259 (46%)	
North America	5, 670 (23%)	4, 153 (21%)	877 (33%)	640 (24%)	
Latin America	1, 916 (7.7%)	1, 626 (8.4%)	94 (3.5%)	196 (7.2%)	
Africa/Middle East	305 (1.2%)	199 (1.0%)	59 (2.2%)	47 (1.7%)	
BMI (kg/m^2^)	27.7 (24.6, 31.5)	27.6 (24.6, 31.6)	28.0 (25.1, 31.6)	27.4 (24.6, 31.1)	<.001
Comorbidities (*n*, %)					
Hypertension	18,687 (75%)	14,265 (73%)	2256 (84%)	2166 (80%)	<.001
Heart failure	5373 (22%)	3564 (18%)	796 (30%)	1013 (37%)	<.001
Diabetes	5725 (23%)	4002 (21%)	808 (30%)	915 (34%)	<.001
Hyperlipidaemia	10,001 (40%)	6685 (34%)	1664 (62%)	1652 (61%)	<.001
LVH	4823 (19%)	3517 (18%)	652 (24%)	654 (24%)	<.001
PAD	712 (2.9%)	323 (1.7%)	178 (6.6%)	211 (7.8%)	<.001
CKD	631 (2.5%)	428 (2.2%)	82 (3.1%)	121 (4.5%)	<.001
Pevious TIA/stroke	3554 (14%)	2714 (14%)	390 (15%)	450 (17%)	.001
COPD	1452 (5.8%)	995 (5.1%)	241 (9.0%)	216 (7.9%)	<.001
Previous bleeding	1307 (5.3%)	933 (4.8%)	186 (6.9%)	188 (6.9%)	<.001
Alcohol use	1663 (6.7%)	1370 (7.0%)	129 (4.8%)	164 (6.0%)	<.001
Smoker	2301 (9.3%)	1765 (9.1%)	233 (8.7%)	303 (11%)	.001
Type of AF (*n*, %)					
Paroxymal AF	13,785 (56%)	10,583 (54%)	1576 (59%)	1626 (60%)	<.001
Persistent AF	8522 (34%)	6782 (35%)	876 (33%)	864 (32%)	
Permanent AF	2520 (10%)	2068 (11%)	225 (8.4%)	227 (8.4%)	
EHRA III‐IV	6, 234 (27%)	4, 758 (26%)	724 (29%)	752 (30%)	<.001
Systolic blood pressure (mmHg)	130 (120, 142)	130 (120, 142)	130 (120, 141)	130 (119, 142)	<.001
Diastolic blood pressure (mmHg)	80 (70, 85)	80 (70, 86)	77 (70, 82)	76 (69, 82)	<.001
Heart rate (bpm)	76 (65, 90)	76 (65, 90)	73 (64, 86)	74 (65, 87)	<.001
Creatinine (umol/L)	76 (58, 99)	77 (59, 100)	73 (56, 93)	71 (52, 93)	<.001
CHA_2_DS_2_‐VASc score	3 (2, 4)	3 (2, 4)	3 (2, 4)	4 (3,5)	<.001
CHA_2_DS_2_‐VASc score ≥2 (*n*, %)	21,254 (86%)	16,184 (83%)	2466 (92%)	2604 (96%)	<.001
HAS‐BLED score	1 [1, 2]	1 [1, 2]	2 [1, 2]	2 [1, 3]	<.001
HAS‐BLED score ≥3 (*n*, %)	3745 (15%)	2507 (13%)	542 (20%)	696 (26%)	<.001
Medications (*n*, %)					
Aspirin alone	2025 (8.2%)	1282 (6.6%)	260 (9.7%)	483 (18%)	<.001
OAC	21,478 (87%)	17,012 (88%)	2331 (87%)	2135 (79%)	<.001
OAC + aspirin	3, 232 (13%)	1, 638 (8.4%)	709 (26%)	885 (33%)	<.001
NOACs	16,751 (67%)	13,354 (69%)	1857 (69%)	1540 (57%)	<.001
Dabigatran	8392 (34%)	6749 (35%)	918 (34%)	725 (27%)	
Rivaroxaban	3810 (15%)	3052 (16%)	405 (15%)	353 (13%)	
Apixaban	4254 (17%)	3300 (17%)	512 (19%)	442 (16%)	
Edoxaban	295 (1.2%)	253 (1.3%)	22 (.8%)	20 (.7%)	
Beta blockers	15,621 (63%)	11,770 (61%)	1843 (69%)	2008 (74%)	<.001
Class III AAD	3709 (15%)	2774 (14%)	463 (17%)	472 (17%)	<.001
Digoxin	2044 (8.2%)	1616 (8.3%)	223 (8.3%)	205 (7.5%)	.4
ACEI	17,269 (70%)	14,009 (72%)	1690 (63%)	1570 (58%)	<.001
ARB	18,438 (74%)	14,454 (74%)	1963 (73%)	2021 (74%)	.5
Stains	11,038 (44%)	7052 (36%)	1835 (69%)	2151 (79%)	<.001
Diuretics	9328 (38%)	6974 (36%)	1141 (43%)	1213 (45%)	<.001

Abbreviations: AAD, antiarrhythmic drug; ACEI, angiotensin converting enzyme inhibitor; AF, atrial fibrillation; ARB, angiotensin receptor blocker; BMI, body mass index; CKD, chronic kidney disease; COPD, chronic obstructive pulmonary disease; LVH, left ventricular hypertrophy; MI, myocardial infarction; NOACs, non‐vitamin K antagonist oral anticoagulant drug; OAC, oral anticoagulants; PAD, peripheral arterial disease; TIA, transient ischemic attack.

The median CHA_2_DS_2_‐VASc and HAS‐BLED scores of the whole cohort were 3 [2, 4] and 1 [1, 2], respectively, but the proportions with CHA_2_DS_2_‐VASc score ≥2 and HAS‐BLED score ≥3 were significantly higher in Group 1 and in Group 2, compared with control group (all *p* < .001). EHRA III‐IV was more common in Group 1 (30%) and Group 2 (29%) than in the Control group (26%) (*p* < .001). Overall, OAC prescription rate was 87% in the whole cohort, but patients in Group 1 were less likely to be prescribed with OAC (79%) and NOACs were also less prescribed (57%) compared with the other two groups; instead, aspirin was more commonly prescribed (18%) and the combination of OAC and aspirin was also higher (33%) in this group. Beta blocker, statins, and diuretics were used more in Group 1 and in Group 2 (all *p* < .001).

### 3.2 Outcomes

Table 2 shows the incidence of outcomes in different groups. The primary endpoint in Group 1 (5.99, 95% CI [5.33, 6.71] per 100 patient‐years) and Group 2 (4.04, 95% CI [3.55, 4.70] per 100 patient‐years) were significantly higher than in the Control group (2.79, 95% CI [2.62, 2.96] per 100 patient‐years) (HR = 2.14, 95% CI 1.89, 2.44, *p* < .001 for Group 1 and HR = 1.47, 95% CI 1.26, 1.71, *p* < .001 for Group 2, respectively). The incidences of all‐cause mortality, MACE, MI, and CV death were also highest in Group 1; however, the risk of stroke wasn't statistically different among the three groups.

**TABLE 2:** Incidence of outcomes in patients with and without CAD.

Outcomes	Events (per 100 patient‐years)	HR	95% CI	*p* Value
Primary outcome‐death or stroke				
Control group (*N* = 19,433)	2.79 (2.62, 2.96)	Reference		
Group 1 (N = 2717)	5.99 (5.33, 6.71)	2.14	1.89, 2.44	<.001
Group 2 (*N* = 2677)	4.04 (3.55, 4.70)	1.47	1.26, 1.71	<.001
Secondary outcome‐All‐cause mortality and MACE				
All‐cause mortality				
Control group (*N* = 19,433)	2.14 (2.00, 2.30)	Reference		
Group 1 (*N* = 2717)	5.20 (4.59, 5.87)	2.42	2.11, 2.79	<.001
Group 2 (*N* = 2677)	3.35 (2.86, 3.89)	1.56	1.32, 1.84	<.001
MACE				
Control group (*N* = 19,433)	1.81 (1.67, 1.95)	Reference		
Group 1 (*N* = 2717)	5.45 (4.82, 6.14)	3.00	2.60, 3.45	<.001
Group 2 (*N* = 2677)	2.96 (2.50, 3.48)	1.63	1.37, 1.95	<.001
MI				
Control group (*N* = 19,433)	.37 (.31, .44)	Reference		
Group 1 (*N* = 2717)	2.23 (1.83, 2.68)	5.98	4.66, 7.67	<.001
Group 2 (*N* = 2677)	.83 (.60, 1.12)	2.24	1.58, 3.16	<.001
CV death				
Control group (*N* = 19,433)	.87 (.78, .97)	Reference		
Group 1 (*N* = 2717)	2.99 (2.53, 3.51)	3.43	2.83, 4.16	<.001
Group 2 (*N* = 2677)	1.57 (1.25, 1.96)	1.81	1.42, 2.31	<.001
Stroke				
Control group (*N* = 19,433)	.90 (.80–1.00)	Reference		
Group 1 (N = 2717)	1.17 (.90, 1.52)	1.31	.99, 1.72	.056
Group 2 (*N* = 2677)	1.11 (.84, 1.44)	1.23	.93, 1.64	.15
Safety endpoint‐major bleeding				
Control group (*N* = 19,433)	.29 (.24, .35)	Reference		
Group 1 (*N* = 2717)	.56 (.37, .80)	1.93	1.28, 2.93	.002
Group 2 (*N* = 2677)	.45 (.29, .68)	1.57	1.00, 2.46	.049

Abbreviations: CV, cardiovascular; MACE, major adverse cardiovascular events; MI, myocardial infarction.

For the safety endpoint, the overall risk of major bleeding was highest in Group 1, followed by Group 2 compared with the Control group (HR = 1.93, 95% CI 1.28, 2.93, *p* = .002 for Group 1 and HR = 1.57, 95% CI 1.00, 2.46, *p* = .049 for Group 2, respectively).

**FIGURE 1:**
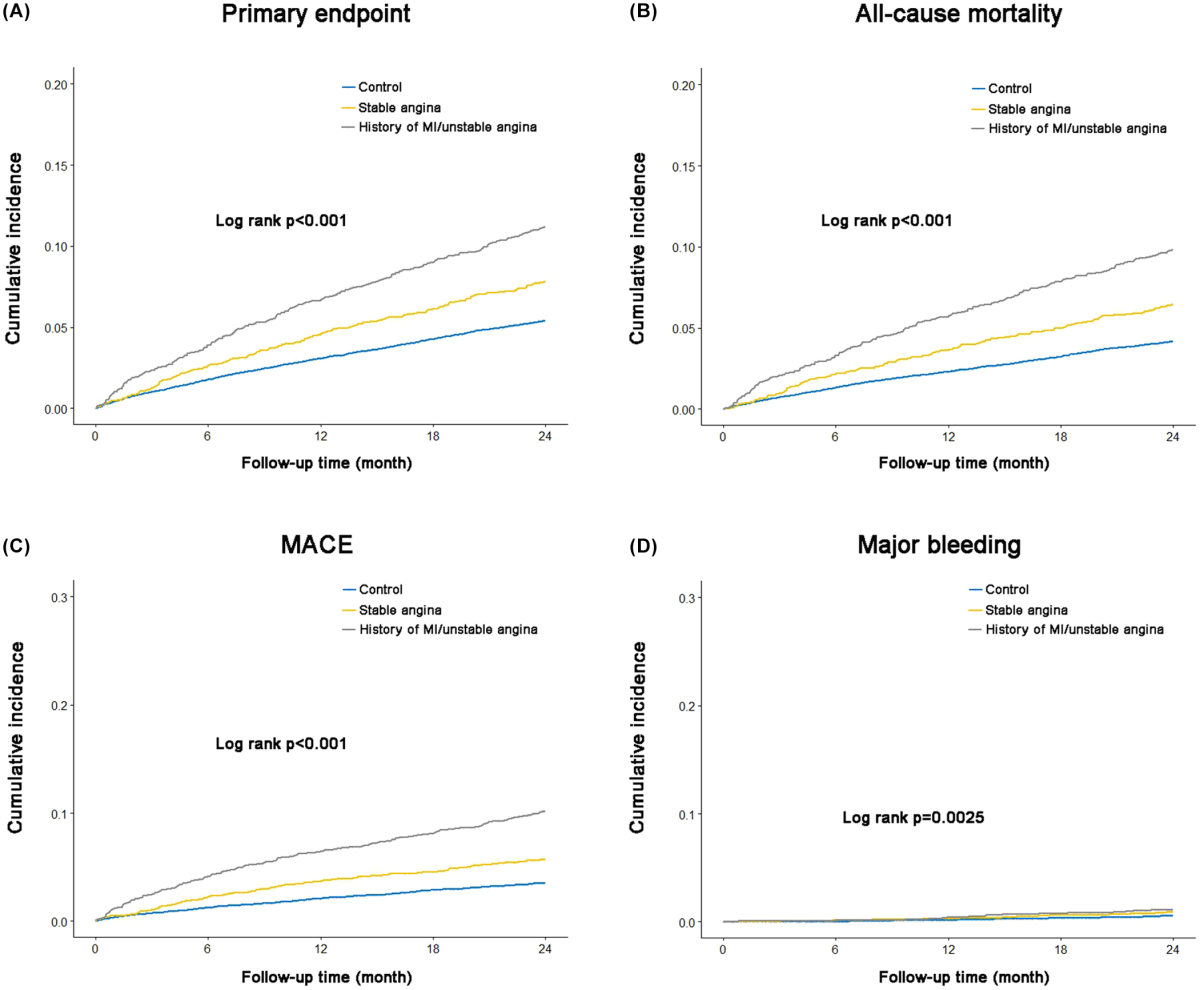
Cumulative incidence of the endpoints in patients with different clinical phenotypes of CAD and those without CAD. (A) K‐M curves for primary endpoint; (B) K‐M curves for all‐cause mortality; (C) K‐M curves for MACE; (D) K‐M curves for major bleeding. MACE, major adverse cardiovascular events.

Figure 1 shows the K‐M curves of each endpoint in the three patient groups. The cumulative incidences of the primary endpoint, all‐cause mortality, MACE, and major bleeding were the highest in Group 1, followed by Group 2 and the control group (all log rank *p* < .05).

### 3.3 Multivariate analysis

**FIGURE 2:**
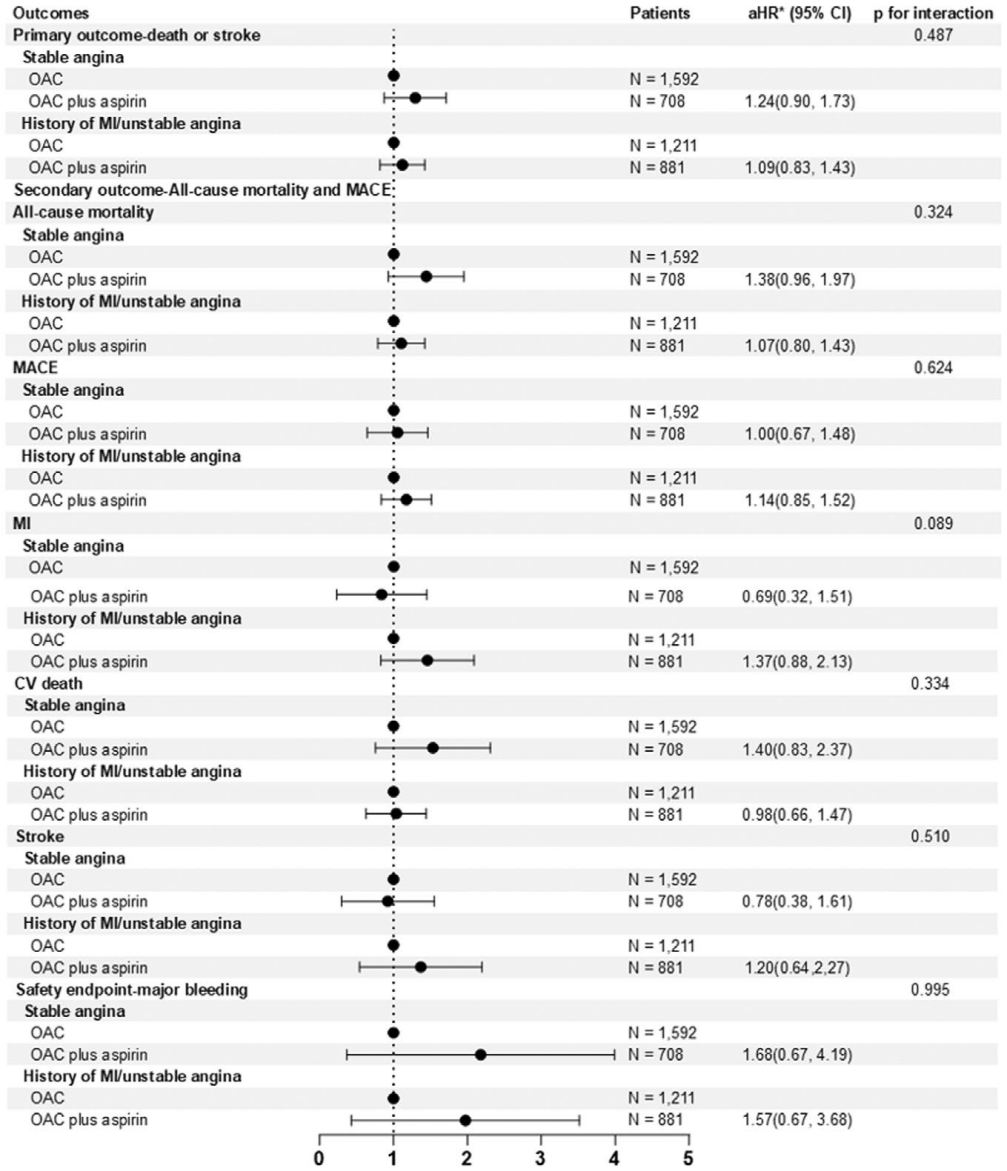
Outcomes between OAC and OAC plus aspirin. CV, cardiovascular; MACE, major adverse cardiovascular events; MI, myocardial infarction; OAC, oral anticoagulants.

Compared with patients in control group, patients in Group 1 and in Group 2 had significantly elevated risks of the primary composite endpoint (aHR = 1.58, 95% CI, 1.37, 1.83, *p* < .001 in Group 1 and aHR = 1.22, 95% CI, 1.04, 1.43, *p* = .012 in Group 2, respectively). The risks of all‐cause mortality, MACE, MI and CV death were increased in Group 2 and were the highest in Group 1. The risks of stroke and major bleeding were comparable among the three groups (Table 3, Tables S1–S4). Figure 2 compares the risk of the various outcomes in patients who received a combination of OAC and aspirin with those who received aspirin alone. For all outcomes, OAC plus aspirin versus OAC monotherapy had comparable risk for all outcomes. No significant interaction between phenotypes of CAD and antithrombotic strategies were observed (all *p* > .05).

**TABLE 3:** Multivariate analysis of impact of different clinical phenotypes of coronary artery disease on the outcome.

Outcomes	aHR^a^	95% CI	*p* Value
Primary outcome‐death or stroke			
Control group (*N* = 19,433)	Reference		
Group 1 (*N* = 2717)	1.58	1.37, 1.83	<.001
Group 2 (*N* = 2677)	1.22	1.04, 1.43	.012
Secondary outcome‐All‐cause mortality and MACE			
All‐cause mortality			
Control group (*N* = 19,433)	Reference		
Group 1 (*N* = 2717)	1.79	1.52, 2.09	<.001
Group 2 (*N* = 2677)	1.30	1.09, 1.54	.004
MACE			
Control group (*N* = 19,433)	Reference		
Group 1 (*N* = 2717)	2.17	1.85, 2.55	<.001
Group 2 (*N* = 2677)	1.33	1.10, 1.60	.003
MI			
Control group (N = 19,433)	Reference		
Group 1 (N = 2717)	4.35	3.24, 5.83	<.001
Group 2 (N = 2677)	1.79	1.25, 2.57	.002
CV death			
Control group (N = 19,433)	Reference		
Group 1 (N = 2717)	2.46	1.97, 3.08	<.001
Group 2 (N = 2677)	1.55	1.20, 2.01	<.001
Stroke			
Control group (*N* = 19,433)	Reference		
Group 1 (*N* = 2717)	1.01	.75, 1.36	>.9
Group 2 (*N* = 2677)	1.06	.79, 1.43	.7
Safety endpoint‐major bleeding			
Control group (*N* = 19,433)	Reference		
Group 1 (*N* = 2717)	1.26	.80, 2.00	.3
Group 2 (*N* = 2677)	1.09	.68, 1.75	.7

Abbreviations: CV, cardiovascular; MACE, major adverse cardiovascular events; MI, myocardial infarction.

^a^ Adjusted for age, sex, body mass index, comorbidities (hypertension, heart failure, left ventricular hypertrophy, diabetes, chronic obstructive pulmonary disease, previous transient ischemic attack/stroke), type of AF, EHRA score, creatinine, systolic blood pressure, heart rate, CHA2DS2‐VASc score, HAS‐BLED score, and medications (aspirin, NOACs [dabigatran, rivaroxaban, apixaban, edoxaban], VKA, beta blockers, digoxin, angiotensin converting enzyme inhibitor, angiotensin receptor blocker, statins, and diuretics).

### 3.4 NOACs versus VKA

**FIGURE 3:**
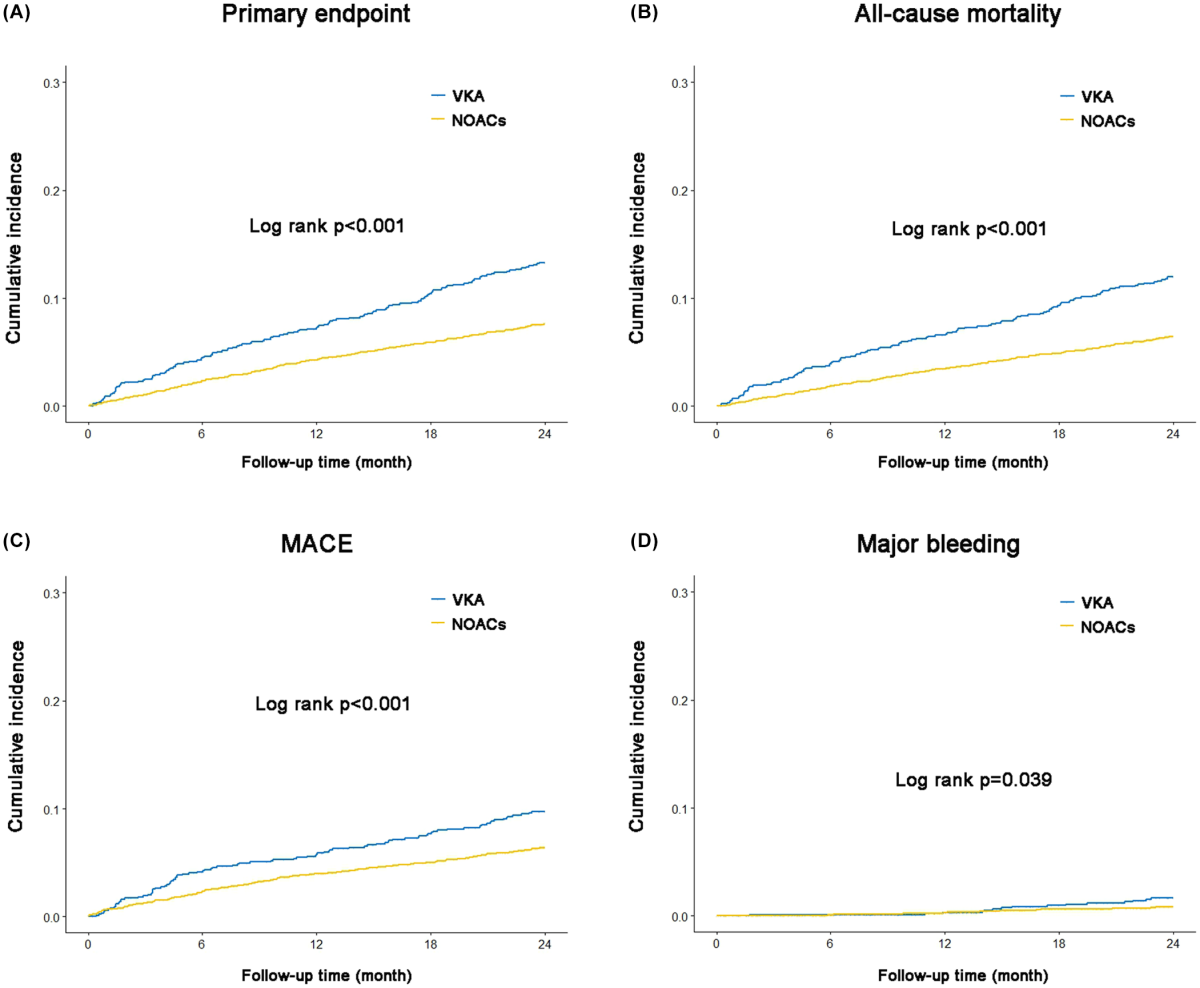
Cumulative incidence of the endpoints between patients received VKA and those with NOACs. (A) K‐M curves for primary endpoint; (B) K‐M curves for all‐cause mortality; (C) K‐M curves for MACE; (D) K‐M curves for major bleeding. MACE, major adverse cardiovascular events; NOACs, non‐vitamin K antagonist oral anticoagulants; VKA, vitamin K antagonists.

Baseline characteristics between patients taking NOACs and those received VKA are shown in Table 4. The K‐M curves between patients received NOACs and those received VKA were shown in Figure 3. The cumulative incidence of the primary endpoint, all‐cause mortality, MACE, and major bleeding in patients received VKA were higher than in patients received NOACs (all log rank *p* < .05).

**TABLE 4:** Baseline characteristics between patients received VKA and received NOACs.

	VKA group (*N* = 995)	NOACs group (*N* = 3, 397)	*p* Value
Age (years)	73 (67, 79)	73 (67, 79)	>.9
Sex (male, %)	645 (65%)	2244 (66%)	.5
BMI (kg/m^2^)	27.7 (24.8, 31.2)	28.3 (25.4, 32.0)	.001
Comorbidities (*n*, %)			
Hypertension	817 (82%)	2847 (84%)	.2
Heart failure	394 (40%)	1088 (32%)	<.001
Diabetes	341 (34%)	1068 (31%)	.092
Hyperlipidaemia	594 (60%)	2276 (67%)	<.001
LVH	292 (29%)	833 (25%)	.002
PAD	71 (7.1%)	257 (7.6%)	.7
CKD	84 (8.4%)	64 (1.9%)	<.001
Pevious TIA/stroke	140 (14%)	531 (16%)	.2
COPD	92 (9.2%)	304 (8.9%)	.8
Previous bleeding	57 (5.7%)	230 (6.8%)	.2
Alcohol use	58 (5.8%)	184 (5.4%)	.6
Smoker	95 (9.5%)	291 (8.6%)	.3
Type of AF (*n*, %)			
Paroxymal AF	489 (49%)	1993 (59%)	<.001
Persistent AF	383 (38%)	1116 (33%)	
Permanent AF	123 (12%)	288 (8.5%)	
Systolic blood pressure (mmHg)	130 (119, 140)	130 (120, 142)	.016
Diastolic blood pressure (mmHg)	75 (68, 82)	77 (70, 82)	.077
Heart rate (bpm)	76 (65, 88)	73 (64, 86)	.031
Creatinine (umol/L)	67 (48, 89)	73 (56, 95)	<.001
CHA_2_DS_2_‐VASc score	4 (3, 5)	4 (3, 5)	.012
CHA_2_DS_2_‐VASc score ≥2 (*n*, %)	960 (96%)	3211 (95%)	.013
HAS‐BLED score	2 (1, 2)	2 (1, 2)	.042
HAS‐BLED score ≥3 (*n*, %)	221 (22%)	669 (20%)	.082
Medications (*n*, %)			
Aspirin	376 (38%)	1213 (36%)	.2
Beta blocker	760 (76%)	2484 (73%)	.040
Class III AAD	194 (19%)	604 (18%)	.2
Digoxin	110 (11%)	258 (7.6%)	<.001
ACEI	569 (57%)	1959 (58%)	.8
ARB	739 (74%)	2504 (74%)	.7
Stains	738 (74%)	2495 (73%)	.6
Diuretics	497 (50%)	1538 (45%)	.009

Abbreviations: AAD, antiarrhythmic drug; ACEI, angiotensin converting enzyme inhibitor; AF, atrial fibrillation; ARB, angiotensin receptor blocker; BMI, body mass index; CKD, chronic kidney disease; COPD, chronic obstructive pulmonary disease; LVH, left ventricular hypertrophy; NOACs, non‐vitamin K antagonist oral anticoagulant drug; PAD, peripheral arterial disease; VKA, vitamin K antagonist.

The results of multivariate‐adjusted Cox regression comparing VKA and NOACs were shown in Table 5. Compared with VKA, NOACs were associated with decreased risk of the primary outcome (aHR = .56, 95% CI, .45, .70, *p* < .001), all‐cause mortality (aHR = .54, 95% CI, .43, .67, *p* < .001), MACE (aHR = .65, 95% CI, .51, .83, *p* < .001), CV death (aHR = .56, 95% CI, .41, .78, *p* < .001), and major bleeding (aHR = .48, 95% CI, .26, .92, *p* = .026), but the risks of MI (aHR = .72, 95% CI, .48, 1.08, *p* = .11) and stroke (aHR = .81, 95% CI, .49, 1.34, *p* = .4) were not significantly different between NOACs and VKA.

**TABLE 5:** Multivariate adjustment regression in patients with NOACs versus with VKA.

Outcomes	aHR^a^	95% CI	*p* Value
Primary outcome‐death or stroke			
VKA (*N* = 995)	Reference		
NOACs (*N* = 3397)	.56	.45, .70	<.001
Secondary outcome‐All‐cause mortality and MACE			
All‐cause mortality			
VKA (*N* = 995)	Reference		
NOACs (*N* = 3397)	.54	.43, .67	<.001
MACE			
VKA (*N* = 995)	Reference		
NOACs (*N* = 3397)	.65	.51, .83	<.001
MI			
VKA (*N* = 995)	Reference		
NOACs (*N* = 3397)	.72	.48, 1.08	.11
CV death			
VKA (*N* = 995)	Reference		
NOACs (*N* = 3397)	.56	.41, .78	<.001
Stroke			
VKA (*N* = 995)	Reference		
NOACs (*N* = 3397)	.81	.49, 1.34	.4
Safety endpoint			
VKA (*N* = 995)	Reference		
NOACs (*N* = 3397)	.48	.26, .92	.026

Abbreviations: CV, cardiovascular; MACE, major adverse cardiovascular events; MI, myocardial infarction; NOACs, novel oral anticoagulant drugs; VKA, vitamin K antagonists.

^a^ Adjusted for age, sex, body mass index, comorbidities (hypertension, heart failure, left ventricular hypertrophy, diabetes, chronic obstructive pulmonary disease, previous transient ischemic attack/stroke), type of AF, EHRA score, creatinine, systolic blood pressure, heart rate, CHA_2_DS_2_‐VASc score, HAS‐BLED score and medications (aspirin, beta blockers, digoxin, angiotensin converting enzyme inhibitor, angiotensin receptor blocker, statins and diuretics).

**FIGURE 4:**
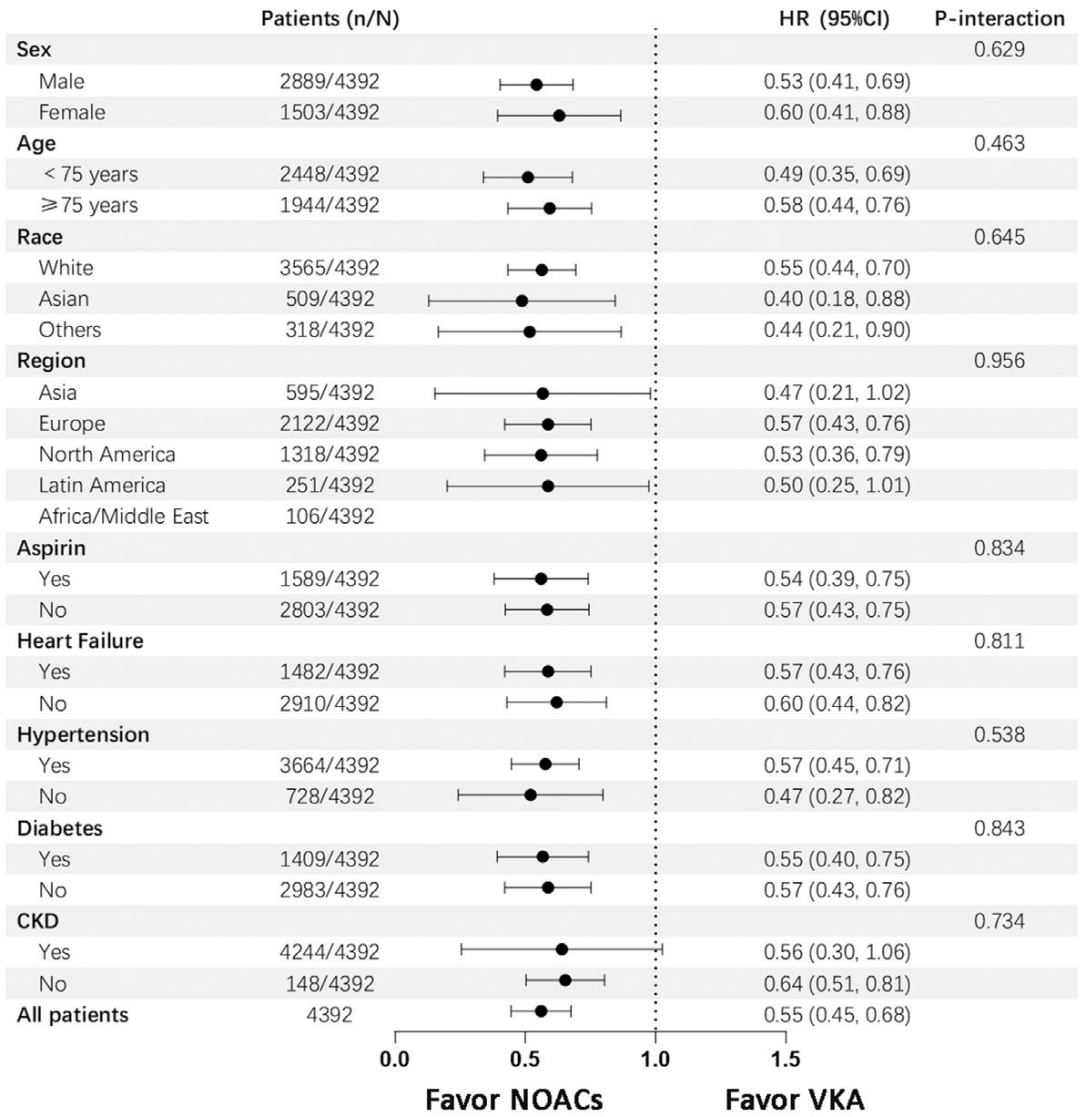
Subgroup analysis of the primary endpoint of NOACs versus VKA. CKD, chronic kidney disease; NOACs, non‐vitamin K antagonist oral anticoagulants; VKA, vitamin K antagonists.

Subgroup analysis of the effect of NOACs versus VKA in different group patients was shown in Figure 4. Compared with VKA, NOACs were associated with reduced risk of the primary endpoint in both male (aHR = .53, 95% CI, .41, .69) and female (aHR = .60, 95% CI, .41, .88) (*p* for interaction .629), age >75 years (aHR = .49, 95% CI, .35, .69) and ≤75 years (aHR = .58, 95% CI, .44, .76) (*p* for interaction .463), white patients (aHR = .55, 95% CI, .44, .70), Asian (aHR = .40, 95% CI, .18, .88) and other races (aHR = .44, 95% CI, .21, .90) (*p* for interaction .645), concomitant use of aspirin (aHR = .54, 95% CI, .39, .75) or not (aHR = .57, 95% CI, .43, .75) (*p* for interaction .834). The effect of NOAC on the primary endpoint was consistent in all subgroups of patients, and NOACs was favoured over VKA across different subgroups. (all interaction *p* > .05).

### 3.5 Sensitivity analysis

To validate the robustness of the findings that NOACs was superior to VKA in patients with AF and CAD, PSM was used to balance the baseline characteristics of the two groups (Table S5). After PSM and compared with VKA, NOACs was significantly associated with reduced risk of the primary endpoint (HR = .68, 95% CI .51, .92, *p* = .011) and all‐cause mortality (HR = .64, 95% CI .47, .88) while there were no statistically significant differences between the two groups in terms of MACE (HR = .86, 95% CI, .62, 1.18, *p* = .403) and major bleeding (HR = .82, 95% CI, .34, 1.97, *p* = .761) (Figure S1).

## 4 DISCUSSION

In this study, almost one quarter of AF patients had concomitant CAD in the GLORIA‐AF registry. Second, CAD was associated with increased risk of poor outcome in patients with AF, and there were prognostic differences among CAD patients with different clinical phenotypes in AF patients, whereby patients with history of prior MI/unstable angina had a worse prognosis compared to those with stable angina. Third, for AF patients with concurrent CAD, the use of NOACs compared to VKA was associated with a significantly reduced primary endpoint and CV events. The effect was consistent in different sexes, races, OAC with and without antiplatelet agent, etc. This study highlights the importance of classifying CAD patients based on clinical phenotypes for prognosis assessment and optimizing the choice of anticoagulant therapy in AF patients with CAD.

Previous studies have shown that CAD and AF often coexist and can have a bidirectional causal relationship, therefore aggravating each other in a vicious circle.^13^ On one hand, ischemia of the atrial tissue in the setting of CAD causes local inflammation, fibrosis, and electrophysiological conduction delays, all of which trigger and promote the occurrence of AF.^14^ AF can also accelerate atherosclerosis through mechanisms such as endothelial dysfunction and inflammation.^15^ Additionally, thrombus formed during AF may directly cause an acute coronary syndrome.^16^

The prevalence of CAD in patients with AF ranges from 17% to 46.5%^3^ and in our study, approximately one quarter of AF patients had concurrent CAD. The wide variation in the reported proportion of AF patients with CAD may be due to differences in the study populations, regions, and inclusion criteria. GLORIA‐AF is a global registry, which also showed regional difference in the prevalence of CAD in AF patients, ranging from 15.1% in Latin America to 34.8% in Africa/Middle East.

In this study, we demonstrated that CAD was associated with increased risk of poor prognosis, regardless of the phenotype of CAD, but the risk was more pronounced in patients with a history of MI/unstable angina. Previous studies have shown that patients with a history of MI are at higher risk for recurrent infarction and adverse events,^17^ but the prognosis of AF patients with a past history of MI has been less clear. Our study demonstrated that AF patients with a prior history of MI experienced a significantly higher incidence of cardiovascular events, which may be related to the progression of coronary artery lesions, concurrent heart failure secondary to MI, and other comorbidities in patients with a history of MI. Indeed, our patients with history of MI/unstable angina had higher prevalences of concomitant heart failure, diabetes, and CKD compared with the control group, but after adjusting for these risk factors, history of MI/unstable angina still remained an independent prognostic factor in AF patients, suggesting its independent prognostic value.

Although CAD is an established risk factor for stroke in AF patients, the risk of stroke was statistically nonsignificant between patients with and without CAD in our study. This may be partly due to the high use of anticoagulation in the GLORIA‐AF registry (87%), which reduced the overall risk of stroke, and low event rates could affect the statistical power. However, the multivariate Cox regression analysis revealed a noticeable trend towards an increased risk of stroke in patients with a history of MI/unstable angina compared to the control group, although this did not reach statistical significance.

The antithrombotic therapy of AF with concomitant CAD can be challenging due to the increased risks of both embolism and bleeding, especially the latter, which is significantly increased when antiplatelet drugs are used concurrently.^18^ Previous meta‐analysis of randomized trials have shown that in AF patients with CAD, NOACs did not significantly increase the risk of embolism compared to VKA.^19^ In contrast, the results of our study indicated that NOACs were superior to VKA in terms of the primary endpoint, death or stroke and were also significantly associated with reduce risks of all‐cause mortality, MACE, and CV death. Regarding safety, NOACs were also associated with reduced the risk of major bleeding by 50% compared to VKA. Of note, NOACs were not associated with increased risk of stroke or MI, and there was even a trend towards risk reduction, aligning with previous research findings.^19^, ^20^, ^21^ Moreover, this advantage of NOACs was consistent across different subgroups, including different sexes, races and regions, etc., suggesting that for AF patients with CAD, NOACs were preferable to VKA in different subgroup patients. In addition, the results of the propensity score analysis showed consistency with the full model regarding the primary endpoint. However, some discrepancies were observed between the propensity score and full model results for MACE and major bleeding. While propensity score analysis helps balance clinical characteristics between groups, it also leads to a reduction in the sample size, potentially influencing statistical power. Nonetheless, for the primary endpoint, the conclusions drawn from both methods were consistent.

Previous studies including the AFIRE trial^22^ and the OAC‐ALONE trial^23^ did not provide robust evidence that anticoagulant monotherapy is superior to a combination of anticoagulants and antiplatelet drug due to the prematurely terminated and (or) non‐standard dosage of OAC. Recent EPIC‐CAD trial showed that in patients with AF combined with stable CAD, edoxaban monotherapy significantly lowered the risk of composite endpoints, including death, embolism and bleeding, particularly major bleeding, compared to edoxaban combined with antiplatelet therapy.^24^ In comparison, our study found that in both stable CAD and previous MI/unstable angina patients, there was no significant increase in the risk of death, embolism or bleeding when using a combination of OAC and antiplatelet drug compared to OAC monotherapy although it revealed the trends of increased risk. However, in terms of primary endpoint, NOACs combined with aspirin was superior to VKA combined with aspirin, demonstrating the safety advantage of NOACs, which is consistent with previous research findings.^25^

GLORIA‐AF had a large, global, multicenter, prospective, real‐world registry design, which had broad representativeness globally and reduced selection bias, and the results reflect real‐world evidence, therefore facilitating the generalization of the findings. While previous research primarily focused on AF patients with established CAD, our study further classified the types of CAD, allowing for a better assessment of the correlations between different CAD phenotypes and the prognosis. Furthermore, the high anticoagulation rate among in this study reflect more standardized treatment practices to some extent, which helps provide a more accurate analysis of risk factors and their relationship with the prognosis.

### 4.1 Limitations

Some limitations in our present study should be addressed. First, although CAD patients were classified based on the clinical phenotype, information regarding the severity of CAD and other treatment measures beyond medication, such as stent implantation or coronary artery bypass grafting, were lacking. Second, CAD types represent a dynamic process, but we only classified patients based on their status at the time of enrollment, and the potential impact of these dynamic changes over time cannot be ignored. Third, in NOACs era, the majority of patients in this study used NOACs, and the proportion of warfarin users was relatively low, resulting in a limited sample size for the warfarin group when comparing NOACs with warfarin. Lastly, this study is a post‐hoc analysis of the GLORIA‐AF registry study, and the interpretation and generalization of the results should be approached with caution.

## 5 CONCLUSION

CAD was prevalent in patients with AF, and clinical phenotypes of CAD influenced outcomes in patients with AF, with history of MI/unstable angina being associated with significantly increased risk of CV events, compared to stable angina. NOACs were superior to VKA in terms of the effectiveness and safety outcomes in patients with AF and concomitant CAD.

## AUTHOR CONTRIBUTIONS

Bi Huang, Yang Liu, Ho Man Lam, Hironori Ishiguchi and Gregory Y.H. Lip designed the study. Bi Huang analysed and wrote the draft. Steven H.M. Lam and Hironori Ishiguchi contributed to the interpretation of the data. Tze F. Chao, Menno V. Huisman and Gregory Y.H. Lip contributed to the revision of the manuscript. All authors read and approved the final manuscript.

## FUNDING INFORMATION

Boehringer Ingelheim GmbH.

## CONFLICT OF INTEREST STATEMENT

Tze F. Chao reports honoraria for lectures from Boehringer Ingelheim, Bayer, Pfizer, and Daiichi Sankyo, outside the submitted work. Menno V. Huisman reports receiving research grants from the Dutch Healthcare Fund, Dutch Heart Foundation, BMS‐Pfizer, Bayer Healthcare and Boehringer Ingelheim, and consulting fees from BMSPfizer, Bayer Healthcare and Boehringer Ingelheim to the institution. Gregory Y.H. Lip is a consultant and speaker for BMS/Pfizer, Boehringer Ingelheim, Daiichi‐Sankyo and Anthos. No fees are received personally. He is a senior investigator at National Institute for Health and Care Research (NIHR) and co‐PI of the AFFIRMO project on multimorbidity in AF (grant agreement number: 899871), TARGET project on digital twins for personalized management of atrial fibrillation and stroke (grant agreement number: 101136244) and ARISTOTELES project on artificial intelligence for management of chronic long‐term conditions (grant agreement number: 101080189), which are all funded by the EU's Horizon Europe Research & Innovation programme. Other authors declare no competing interests.

## Supporting information


Figure S1.

Table S1.

## Data Availability

Data supporting this study by the data contributors Boehringer Ingelheim and were made and are available through Vivli, Inc. Access was provided after a proposal was approved by an independent review committee identified for this purpose and after receipt of a signed data sharing agreement.
