# Supplementary material for: The impact of clinical phenotypes of coronary artery disease on outcomes in patients with atrial fibrillation: A post‐hoc analysis of GLORIA‐AF registry
Source: Eur J Clin Invest. 2025 Jan 13;55(3):e14378. doi: 10.1111/eci.14378 (PMC11810563; doi:10.1111/eci.14378)
Supplement: Supplementary file 1 — Figure S1. [file ECI-55-e14378-s002.zip › FigureS1.docx]

**Supplement Figure 1:** Cumulative incidence of the endpoints between patients received VKA and those with NOACs after PSM. (A) K-M curves for primary endpoint; (B) K-M curves for all-cause mortality; (C) K-M curves for MACE; (D) K-M curves for major bleeding. MACE, major adverse cardiovascular events; VKA, vitamin K antagonists; NOACs, non-vitamin K antagonist oral anticoagulants
