# Supplementary figures and images for: The impact of clinical phenotypes of coronary artery disease on outcomes in patients with atrial fibrillation: A post‐hoc analysis of GLORIA‐AF registry
Source: Eur J Clin Invest. 2025 Jan 13;55(3):e14378. doi: 10.1111/eci.14378 (PMC11810563; doi:10.1111/eci.14378)

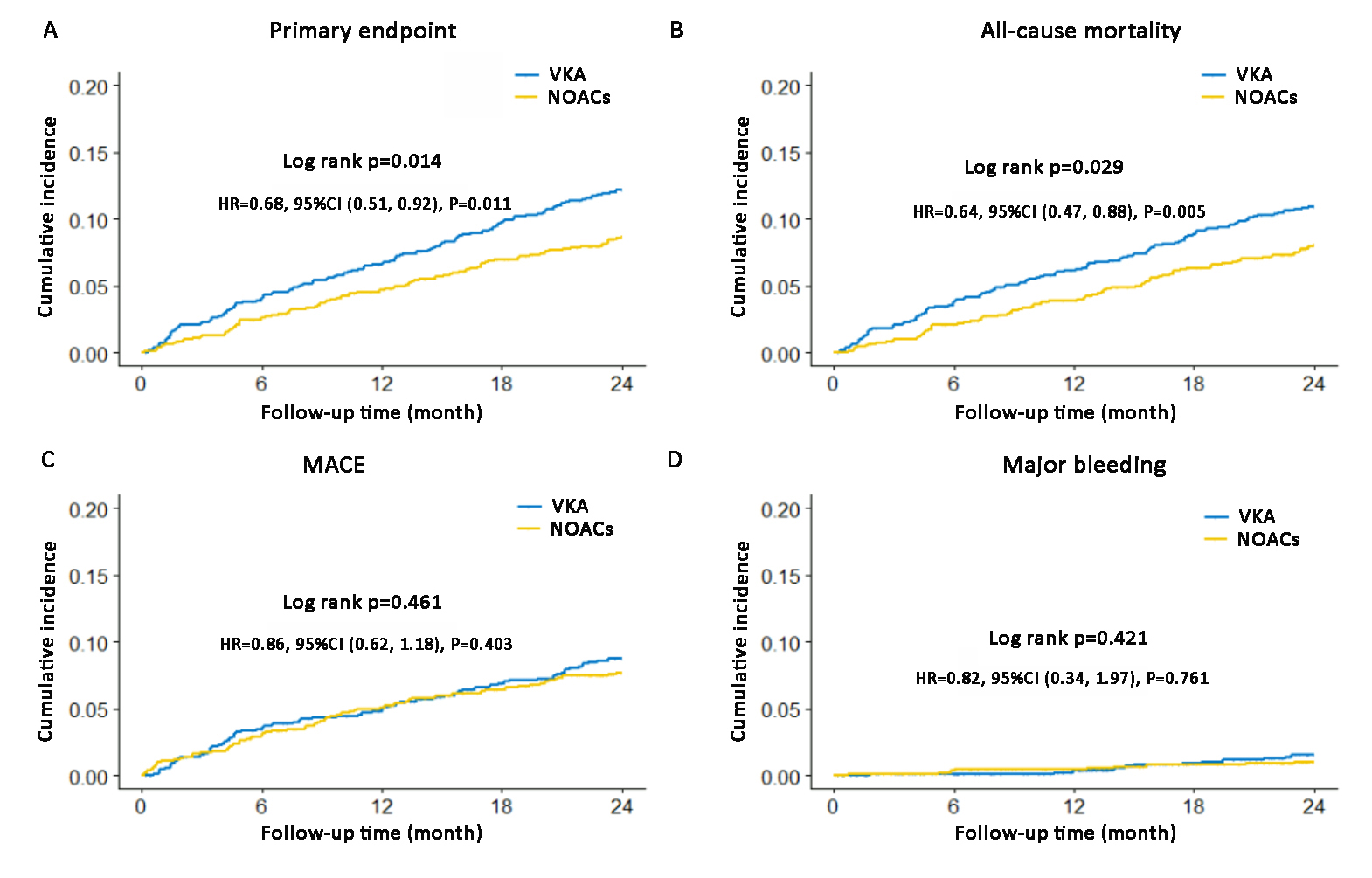

Supplement: Supplementary file 1 — Figure S1. [file ECI-55-e14378-s002.zip › eci14378-sup-0001-FigureS1.jpg]
