# Supplementary material for: The impact of clinical phenotypes of coronary artery disease on outcomes in patients with atrial fibrillation: A post‐hoc analysis of GLORIA‐AF registry
Source: Eur J Clin Invest. 2025 Jan 13;55(3):e14378. doi: 10.1111/eci.14378 (PMC11810563; doi:10.1111/eci.14378)
Supplement: Supplementary file 2 — Table S1. [file ECI-55-e14378-s001.zip › eci14378-sup-0002-TableS1.docx]

**Supplement table 1.** Factors associated with primary outcome with multivariate analysis

| **Variables** | **HR*** | **95% CI** | **p value** |
| --- | --- | --- | --- |
| CAD status |  |  |  |
| Control group | Reference |  |  |
| Group 1 | 1.58 | 1.37, 1.83 | <0.001 |
| Group 2 | 1.22 | 1.04, 1.43 | 0.012 |
| Female | 0.74 | 0.67, 0.83 | <0.001 |
| Age (≥75 years) | 2.10 | 1.86, 2.36 | <0.001 |
| SBP at enrollment | 0.74 | 0.66, 0.83 | <0.001 |
| Previous TIA/stroke | 1.60 | 1.41, 1.81 | <0.001 |
| Diabetes | 1.30 | 1.15, 1.45 | <0.001 |
| CHA_2_DS_2_-VASc score≥2 | 1.57 | 1.32, 1.86 | <0.001 |
| Use of ARB | 0.82 | 0.72, 0.94 | 0.003 |
| Use of diuretics | 1.59 | 1.42, 1.77 | <0.001 |
| Use of OAC | 0.64 | 0.55, 0.74 | <0.001 |

CAD, coronary artery disease; SBP, systolic blood pressure; TIA, transient ischemic attack; ARB, angiotensin receptor blocker; OAC, oral anticoagulants

*****Adjusted for age, sex, body mass index, comorbidities (hypertension, heart failure, left ventricular hypertrophy, diabetes, chronic obstructive pulmonary disease, previous transient ischemic attack/stroke), type of AF, EHRA score, creatinine, systolic blood pressure, heart rate, CHA2DS2-VASc score, HAS-BLED score, and medications (aspirin, NOACs [dabigatran, rivaroxaban, apixaban, edoxaban], VKA, beta blockers, digoxin, angiotensin converting enzyme inhibitor, angiotensin receptor blocker, statins, and diuretics).
