# Supplementary material for: The impact of clinical phenotypes of coronary artery disease on outcomes in patients with atrial fibrillation: A post‐hoc analysis of GLORIA‐AF registry
Source: Eur J Clin Invest. 2025 Jan 13;55(3):e14378. doi: 10.1111/eci.14378 (PMC11810563; doi:10.1111/eci.14378)
Supplement: Supplementary file 2 — Table S1. [file ECI-55-e14378-s001.zip › eci14378-sup-0003-TableS2.docx]

**Supplement table 2.** Factors associated with all-cause mortality with multivariate analysis

| **Variables** | **HR*** | **95% CI** | **p value** |
| --- | --- | --- | --- |
| CAD status |  |  |  |
| Control group | Reference |  |  |
| Group 1 | 1.79 | 1.52, 2.09 | <0.001 |
| Group 2 | 1.30 | 1.09, 1.54 | 0.004 |
| Female | 0.73 | 0.64, 0.82 | <0.001 |
| Age (≥75 years) | 2.37 | 2.07, 2.71 | <0.001 |
| SBP at enrollment | 0.66 | 0.58, 0.74 | <0.001 |
| Previous TIA/stroke | 1.42 | 1.23, 1.64 | <0.001 |
| Diabetes | 1.36 | 1.20, 1.55 | <0.001 |
| COPD | 2.04 | 1.73, 2.42 | <0.001 |
| CHA_2_DS_2_-VASc score≥2 | 1.66 | 1.36, 2.02 | <0.001 |
| Use of ARB | 0.82 | 0.70, 0.95 | 0.007 |
| Use of diuretics | 1.80 | 1.59, 2.03 | <0.001 |
| Use of OAC | 0.68 | 0.57, 0.80 | <0.001 |

CAD, coronary artery disease; SBP, systolic blood pressure; TIA, transient ischemic attack; COPD, chronic obstructive pulmonary disease; ARB, angiotensin receptor blocker; OAC, oral anticoagulants

*****Adjusted for age, sex, body mass index, comorbidities (hypertension, heart failure, left ventricular hypertrophy, diabetes, chronic obstructive pulmonary disease, previous transient ischemic attack/stroke), type of AF, EHRA score, creatinine, systolic blood pressure, heart rate, CHA2DS2-VASc score, HAS-BLED score, and medications (aspirin, NOACs [dabigatran, rivaroxaban, apixaban, edoxaban], VKA, beta blockers, digoxin, angiotensin converting enzyme inhibitor, angiotensin receptor blocker, statins, and diuretics).
