# Supplementary material for: The impact of clinical phenotypes of coronary artery disease on outcomes in patients with atrial fibrillation: A post‐hoc analysis of GLORIA‐AF registry
Source: Eur J Clin Invest. 2025 Jan 13;55(3):e14378. doi: 10.1111/eci.14378 (PMC11810563; doi:10.1111/eci.14378)
Supplement: Supplementary file 2 — Table S1. [file ECI-55-e14378-s001.zip › eci14378-sup-0005-TableS4.docx]

**Supplement table 4.** Factors associated with major bleeding with multivariate analysis

| **Variables** | **HR*** | **95% CI** | **p value** |
| --- | --- | --- | --- |
| CAD status |  |  |  |
| Control group | Reference |  |  |
| Group 1 | 1.26 | 0.80, 2.00 | 0.3 |
| Group 2 | 1.09 | 0.68, 1.75 | 0.7 |
| Age (≥75 years) | 1.99 | 1.39, 2.85 | <0.001 |
| Diabetes | 1.62 | 1.16, 2.29 | 0.005 |
| COPD | 2.02 | 1.26, 3.25 | 0.004 |

CAD, coronary artery disease; COPD, chronic obstructive pulmonary disease

*****Adjusted for age, sex, body mass index, comorbidities (hypertension, heart failure, left ventricular hypertrophy, diabetes, chronic obstructive pulmonary disease, previous transient ischemic attack/stroke), type of AF, EHRA score, creatinine, systolic blood pressure, heart rate, CHA2DS2-VASc score, HAS-BLED score, and medications (aspirin, NOACs [dabigatran, rivaroxaban, apixaban, edoxaban], VKA, beta blockers, digoxin, angiotensin converting enzyme inhibitor, angiotensin receptor blocker, statins, and diuretics).
