# Supplementary material for: The impact of clinical phenotypes of coronary artery disease on outcomes in patients with atrial fibrillation: A post‐hoc analysis of GLORIA‐AF registry
Source: Eur J Clin Invest. 2025 Jan 13;55(3):e14378. doi: 10.1111/eci.14378 (PMC11810563; doi:10.1111/eci.14378)
Supplement: Supplementary file 2 — Table S1. [file ECI-55-e14378-s001.zip › eci14378-sup-0006-TableS5.docx]

**Supplement table 5.** Baseline characteristics between patients received VKA and received NOACs before and after PSM

|  | **Before PSM** | | |  | **After PSM** | | |
| --- | --- | --- | --- | --- | --- | --- | --- |
|  | **VKA group**  (N = 995) | **NOACs group**  (N = 3, 397) | **SMD** |  | **VKA group**  **(N = 928)** | **NOACs group**  **(N = 928)** | **SMD** |
| **Age (years)** | 73 (67, 79) | 73 (67, 79) | 0.010 |  | 73 (67, 79) | 72 (66, 78) | 0.059 |
| **Sex (male, %)** | 645 (65%) | 2,244 (66%) | 0.026 |  | 599 (65%) | 612 (66%) | 0.030 |
| **BMI (kg/m^2^)** | 27.7 (24.8, 31.2) | 28.3 (25.4, 32.0) | 0.085 |  | 27.7 (25.0, 31.2) | 28.0 (25.2, 31.7) | 0.010 |
| **Comorbidities (n, %)** |  |  |  |  |  |  |  |
| **Hypertension** | 817 (82%) | 2,847 (84%) | 0.046 |  | 759 (82%) | 772 (83%) | 0.038 |
| **Heart failure** | 394 (40%) | 1,088 (32%) | 0.162 |  | 353 (38%) | 347 (37%) | 0.014 |
| **Diabetes** | 341 (34%) | 1,068 (31%) | 0.061 |  | 310 (33%) | 316 (34%) | 0.014 |
| **Hyperlipidaemia** | 594 (60%) | 2,276 (67%) | 0.155 |  | 555 (60%) | 566 (61%) | 0.025 |
| **LVH** | 292 (29%) | 833 (25%) | 0.112 |  | 260 (28%) | 259 (28%) | 0.003 |
| **PAD** | 71 (7.1%) | 257 (7.6%) | 0.016 |  | 63 (6.8%) | 52 (5.6%) | 0.045 |
| **CKD** | 84 (8.4%) | 64 (1.9%) | 0.482 |  | 25 (2.7%) | 31 (3.3%) | 0.048 |
| **Pevious TIA/stroke** | 140 (14%) | 531 (16%) | 0.043 |  | 126 (14%) | 122 (13%) | 0.012 |
| **COPD** | 92 (9.2%) | 304 (8.9%) | 0.010 |  | 87 (9.4%) | 95 (10%) | 0.030 |
| **Previous bleeding** | 57 (5.7%) | 230 (6.8%) | 0.042 |  | 55 (5.9%) | 61 (6.6%) | 0.026 |
| **Alcohol use** | 58 (5.8%) | 184 (5.4%) | 0.018 |  | 58 (5.9%) | 61 (6.6%) | 0.029 |
| **Smoker** | 95 (9.5%) | 291 (8.6%) | 0.035 |  | 89 (9.6%) | 82 (8.8%) | 0.027 |
| **Type of AF (n, %)** |  |  | 0.207 |  |  |  | 0.003 |
| **Paroxymal AF** | 489 (49%) | 1,993 (59%) |  |  | 467 (50%) | 471 (51%) |  |
| **Persistent AF** | 383 (38%) | 1,116 (33%) |  |  | 349 (38%) | 343 (37%) |  |
| **Permanent AF** | 123 (12%) | 288 (8.5%) |  |  | 112 (12%) | 114 (12%) |  |
| **Systolic blood pressure (mmHg)** | 130 (119, 140) | 130 (120, 142) | 0.084 |  | 130 (120, 140) | 130 (120, 141) | 0.023 |
| **Diastolic blood pressure (mmHg)** | 75 (68, 82) | 77 (70, 82) | 0.064 |  | 76 (69, 83) | 77 (70, 82) | 0.051 |
| **Heart rate (bpm)** | 76 (65, 88) | 73 (64, 86) | 0.069 |  | 76 (65, 88) | 74 (65, 88) | 0.003 |
| **Creatinine (umol/L)** | 67 (48, 89) | 73 (56, 95) | 0.199 |  | 69 (52, 90) | 72 (55, 92) | 0.053 |
| **CHA_2_DS_2_-VASc score** | 4 (3, 5) | 4 (3, 5) | 0.124 |  | 4 (3, 5) | 4 (3, 5) | 0.018 |
| **CHA_2_DS_2_-VASc score>=2 (n, %)** | 960 (96%) | 3,211 (95%) | 0.086 |  | 894 (96%) | 883 (95%) | 0.052 |
| **HAS-BLED score** | 2 (1, 2) | 2 (1, 2) | 0.054 |  | 2 (1, 2) | 2 (1, 2) | 0.031 |
| **HAS-BLED score>=3 (n, %)** | 221 (22%) | 669 (20%) | 0.063 |  | 180 (19%) | 182 (20%) | 0.005 |
| **Medications (n, %)** |  |  |  |  |  |  |  |
| **Aspirin** | 376 (38%) | 1,213 (36%) | 0.043 |  | 347 (37%) | 348 (38%) | 0.002 |
| **Beta blocker** | 760 (76%) | 2,484 (73%) | 0.074 |  | 702 (76%) | 724 (78%) | 0.054 |
| **Class III AAD** | 194 (19%) | 604 (18%) | 0.045 |  | 179 (19%) | 182 (20%) | 0.009 |
| **Digoxin** | 110 (11%) | 258 (7.6%) | 0.131 |  | 98 (11%) | 93 (10%) | 0.020 |
| **ACEI** | 569 (57%) | 1,959 (58%) | 0.001 |  | 524 (56%) | 529 (57%) | 0.011 |
| **ARB** | 739 (74%) | 2,504 (74%) | 0.013 |  | 688 (74%) | 685 (74%) | 0.007 |
| **Stains** | 738 (74%) | 2,495 (73%) | 0.016 |  | 688 (74%) | 693 (75%) | 0.017 |
| **Diuretics** | 497 (50%) | 1,538 (45%) | 0.094 |  | 457 (49%) | 447 (48%) | 0.022 |

PSM, propensity score matching; VKA, vitamin K antagonist; NOACs, non-vitamin K antagonist oral anticoagulant drug; BMI, body mass index; LVH, left ventricular hypertrophy; PAD, peripheral arterial disease; CKD, chronic kidney disease; TIA, transient ischemic attack; COPD, chronic obstructive pulmonary disease; AF, atrial fibrillation; AAD, antiarrhythmic drug; ACEI, angiotensin converting enzyme inhibitor; ARB, angiotensin receptor blocker.
